# GDF9 and BMP15 Expressions and Fine Structure Changes During Folliculogenesis in Polycystic Ovary Syndrome

**DOI:** 10.4274/balkanmedj.2016.1110

**Published:** 2018-01-20

**Authors:** Meryem İlkay Karagül, Savaş Aktaş, Banu Coşkun Yılmaz, Mustafa Yılmaz, Gülhan Orekici Temel

**Affiliations:** 1 Department of Histology and Embryology, Mersin University School of Medicine, Mersin, Turkey; 2 Department of Histology and Embryology, University of Health Sciences School of Medicine, İstanbul, Turkey; 3 Department of Biostatistics and Medical Informatics, Mersin University School of Medicine, Mersin, Turkey

**Keywords:** Polycystic ovary syndrome, folliculogenesis, growth differentiation factor 9, bone morphogenetic protein 15

## Abstract

**Background:**

Polycystic ovary syndrome is the most frequently seen endocrine disorder in women of reproductive age with a prevalence of about 10%.

**Aims:**

To investigate the efficiency of growth differentiation factor 9 and bone morphogenetic protein 15 during folliculogenesis in a dehydroepiandrosterone-induced mouse Polycystic ovary syndrome model.

**Study Design:**

Animal experimentation.

**Methods:**

Mice were divided into 3 groups: control, vehicle and Polycystic ovary syndrome. Polycystic ovary syndrome model mice were developed by the injection of dehydroepiandrosterone dissolved in 0.1 mL of sesame oil. Ovarian tissues were examined for growth differentiation factor 9 and bone morphogenetic protein 15 using immunofluorescent labelling and electron microscopic examinations.

**Results:**

The immunoreactivity of growth differentiation factor 9 and bone morphogenetic protein 15 proteins decreased (p<0.05) in the Polycystic ovary syndrome group (27.73±8.43 and 24.85±7.03, respectively) compared with the control group (33.72±11.22 and 31.12±11.05, respectively) and vehicle group (33.95±10.75 and 29.99±10.72, respectively). Apoptotic changes were observed in granulosa cells, lipid vacuoles increased in Theca cells and thickening and irregularities were noted in the basal lamina of granulosa cells. An increased electron density in the zona pellucida in some of the multilaminar primary and secondary follicles in the Polycystic ovary syndrome model was also observed at the ultrastructural level.

**Conclusion:**

These results suggest that the decrease in the growth differentiation factor 9 and bone morphogenetic protein 15 expression initiated at the primary follicle stage effect the follicle development and zona pellucida structure and may cause subfertility or infertility in Polycystic ovary syndrome.

Polycystic ovary syndrome (PCOS) is the most frequently seen endocrine disorder in women of reproductive age with a prevalence of 10% (1). Ovarian hyperandrogenism, paracrine dysregulation of the follicle, menstrual dysfunction and insulin resistance development in ovaries are typical characteristics of PCOS (2,3).

Folliculogenesis and the menstrual cycle consists of the selection of the dominant follicle, stages of follicular development and ovulation and synchronised endometrial changes (4). Ovarian folliculogenesis is organised by the interaction of extraovarian and intraovarian factors that coordinate the processes of oocyte growth and follicular development in the periovulatory period (5). Primordial follicle growth is primarily influenced by paracrine and endocrine factors (6). Among the many extraovarian and intraovarian factors, the transforming growth factor beta (TGFB) superfamily in particular plays an important role in follicle growth. Two related members of the TGFB superfamily are involved in the function and development of the ovary: Growth differentiation factor 9 (GDF9) and bone morphogenetic protein 15 (BMP15) (7).

BMP15 and GDF9 play a critical role in follicle development, oocyte maturation, ovulation and embryo development (8,9). A study on rats demonstrated that the oocyte-derived GDF9-enhanced passage of primordial follicles to the primary stage was required for pre-antral follicle development (10). In GDF9 null mice, follicle development was arrested at the primary stage with no further growth (11). BMP15 functions are important in the regulation of normal follicular development and ovulation (12). BMP15 is mitogenic for somatic cells and is known to induce granulosa cell proliferation and affect the choice of the dominant follicle as well as the formation of follicular atresia (12).

*In vivo* and *in vitro* studies have suggested that GDF9 and BMP15 contribute to the formation of the pathogenesis of PCOS (13,14,15). However, studies investigating the roles of GDF9 and BMP15 proteins through the stages of folliculogenesis in the pathogenesis of PCOS do not currently exist. Therefore, in the present study, we investigated the involvement of GDF9 and BMP15 in the folliculogenesis process by using a dehydroepiandrosterone (DHEA) exposure mouse model of PCOS.

## MATERIALS AND METHODS

### Animals and experimental design

Forty five female prepubertal (23-24 days old) mice of the BALB/c were used in the this study. The mice were holded in polycarbonate boxes and maintained in 12-hour light/12-hour dark cycle, and 50%-70% humidity, 23±1 °C temperature, with free access to water and food. The animals were randomly divided into 3 groups each consisting of 15 mice: control group (no injections were performed to mice); vehicle group [mice were injected subcutaneously (SC) with 0.1 mL of sesame oil (Sigma-Aldrich, İstanbul, Turkey) daily for 20 consecutive days] and PCOS group [DHEA (dissolved in 0.1 mL sesame oil, 6 mg/100 g body weight; Merck Millipore, İstanbul, Turkey) was injected to each mice for 20 consecutive days SC]. The phases of the estrous cycle for each mice were determined by daily histological examination of vaginal smears. The ethical approval was obtained from Mersin University School of Medicine and its number is 2012-11.

### Tissue preparation methods

Mice were anesthetised by intraperitoneal injections of 10 mg/kg Xylazine Hydrochloride and 90 mg/kg Ketamine Hydrochloride and sacrificed by cervical dislocation at the end of the experiment. Left ovaries of all groups were fixed in 4% paraformaldehyde overnight at 4 °C and then left in 20% sucrose/phosphate buffered saline (PBS) overnight at 4 °C. Afterwards, the tissues were transferred to a 30% sucrose solution with 0.1% sodium azide in PBS and left at 4 °C. Tissues were embedded in cryostat embedding medium (Bio-Optica, Milan, Italy) and sections with 5 µm thickness were cut at -20 °C with a cryostat.

The right ovarian tissues were obtained from mice in each group and cut into 1 mm3 pieces and fixed in 2.5% glutaraldehyde solution for 4-6 hours, and then samples were washed with 0.1 M PBS. After fixation, tissues were postfixed in osmium tetroxide. Then the routine electron microscopy protocol was performed on the tissue and it was embedded in a resin kit.

### Light and electron microscopy

Semi-thin sections (2 µm) and ultra-thin sections (70 nm) were cut by a Leica UCT-125 ultramicrotome (Leica Microsystems GmbH, Wien, Austria). For light microscopic examination of ovaries, semi-thin sections were stained with toluidine blue and then investigated with an Olympus BX50 light microscope (Olympus Corporation, Tokyo, Japan). For electron microscopic examination of ovarian stromal and follicular cells, ultra-thin sections contrasted with uranyl acetate and lead citrate were examined by transmission electron microscopy (Jeol JEM 1011, Tokyo, Japan).

### Immunofluorescence staining

Frozen cryostat sections were washed PBS and then blocked with 5% normal goat serum for GDF9 and with 5% normal rabbit serum for BMP15 in PBS at 37 °C for 30 minutes. Sections were then incubated with the primary antibodies in dilution buffer (PBS containing 0.1% Triton X-100, 1% BSA) overnight at 4 °C as follows: 1) goat polyclonal anti-GDF9 primary antibody (Santa Cruz Biotechnology, CA, USA) at a dilution of 1:100 and 2) rabbit polyclonal anti-BMP15 (GDF9B) primary antibody (Santa Cruz Biotechnology, CA, USA) at a dilution of 1:50. The sections incubated only with PBS were used as a negative control. After being washed in PBS 3 times for 5 minutes, cryosections for GDF9 were incubated with a secondary antibody [tetramethylrhodamine (TRITC) conjugated rabbit anti-goat immunoglobulin G (IgG) antibody, Santa Cruz Biotechnology, CA, USA] at a dilution of 1:50 at room temperature for 1 hour. Cryosections for BMP15 were incubated with a secondary antibody [fluorescein isothiocyanate (FITC) conjugated goat anti-rabbit IgG antibody, Santa Cruz Biotechnology, CA, USA] at a dilution of 1:50 in dilution buffer (PBS containing 0.1% Triton X-100, 1% BSA) at room temperature for 1 hour. They were washed in PBS 3 times for 5 minutes each. Finally, cryosections were mounted in antifade with 4',6-diamidino-2-phenylindole (Santa Cruz Biotechnology, CA, USA).

### Quantitative evaluation of immunofluorescence marking

For staining intensity analysis, sdigital images were taken using a Nikon EclipseTi-S fluorescence microscope (Tokyo, Japan) with a Nikon Digital Sight DS-Fi1c camera. Immunostained images were converted to the 8-bit colour format, and staining intensity was measured using Image J (U. S. National Institutes of Health, Bethesda, Maryland, USA). For GDF9 and BMP15, the mean staining density was determined for the cytoplasm of the granulosa cells and the oocytes of each follicle. In the evaluation, 20 instances of each type of follicle were analysed: Primordial follicles, primary follicles, secondary follicles and tertiary follicles. All of the data were calculated using the pixel brightness value. A higher pixel value indicates stronger immunoreactivity.

### Statistical analysis

In all groups, the variables BMP15 and GDF9 were checked by Shapiro Wilks test whether they satisfy normal distribution conditions. For compare the differences across groups and within the groups, an ANOVA test was implemented. Tukey HSD test was used for pairwise comparison of groups. For a graphical representation of the group distribution, graphs with "Error bar" were preferred. The level of statistical significance is accepted as p<0.05.

## RESULTS

### Vaginal smear findings

We observed changed ovarian function that was affirmed by the lack of the normal regular estrous cycle (proestrous, estrous, metaestrous and diestrous stages). The animals in the control and vehicle groups exhibited normal regular cycling activity. In the PCOS group, irregular cycles occurred after a few days of treatment with DHEA. Estrous stages were restricted to metaestrous or estrous upon DHEA treatment in PCOS group (results not shown).

### Histological examination of mouse ovaries

**Figure 1a-f:**
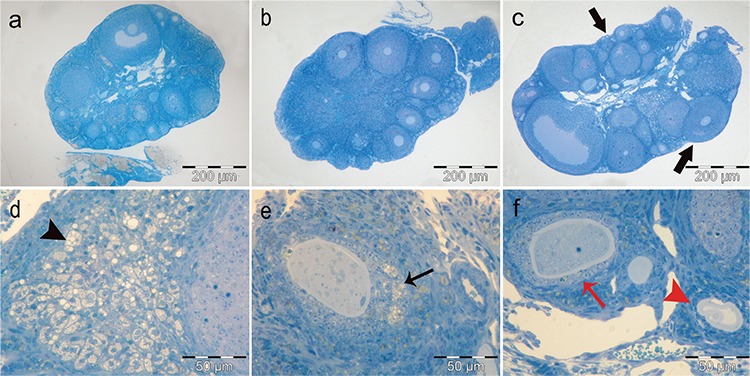
Normal ovarian morphology in control group (a) and vehicle group (b) (x300). Pre-antral follicle numbers increased in the PCOS group (c) (thick arrows) (x300). Luteinisation of granulosa cells (d) (arrowheads) and stromal cells (e) (thin arrow), follicular cysts (f) (red arrows) and atretic follicles (red arrowhead) are seen in PCOS group (x1200) (toluidine blue).

Control group and vehicle group samples show that all follicular stages demonstrate normal histological morphology (Figure 1a, 1b, respectively). In the PCOS group, we observed a raise in the number of pre-antral follicles (Figure 1c). Numerous lipid vacuoles were found in the cytoplasm of granulosa cells in some ovarian follicles and in the cytoplasm of stromal cells (Figure 1d, 1e, respectively). In addition, atretic follicles and cystic follicles of different sizes were observed. (Figure 1f).

### Immunofluorescence findings

**Figure 2a,b:**
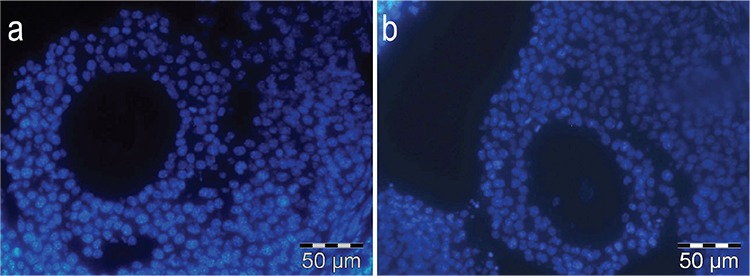
Immunofluorescence negative controls of GDF9 (a) and BMP15 (b) (x1200).

There was no immunolabelling in the negative immunofluorescence controls for GDF9 and BMP15 (Figure 2a, 2b, respectively).

### Immunolabelling of GDF9

**Figure 3a-d:**
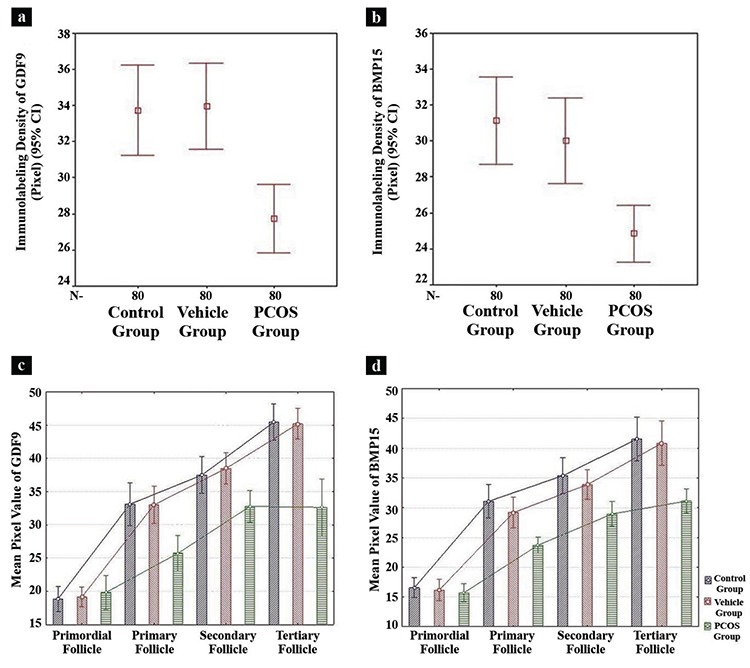
Comparison between groups of immunolabelling density of GDF9 (a) and BMP15 (b). Graphical comparison of immunolabelling intensity of GDF9 (c) and BMP15 (d) in stages of follicular development. *CI: confidence interval; PCOS: polycystic ovary syndrome; GDF9: growth differentiation factor 9; BMP15: bone morphogenetic protein 15; Bars represent mean ± standard deviation values.*

**Figure 4a-l:**
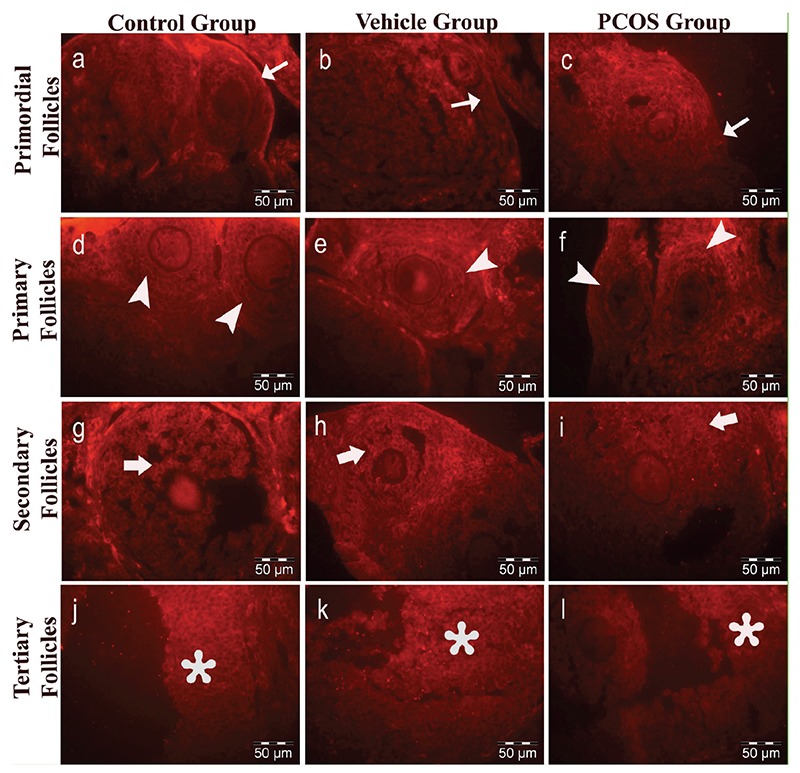
Immunolabelling intensity of GDF9. Control group: a, d, g, j; vehicle group: b, e, h, k; and PCOS group: c, f, i, l. Labelling within a-c: primordial (thin arrow); d-f: multilaminar primary (arrowheads); d-i: secondary (thick arrow) and j-l: tertiary follicles (asterisk) (x1200, TRITC).

GDF9 immunoreactivity in the control group (33.72±11.22) and vehicle group (33.95±10.75; Table 1, Figure 3a) was similar. In both groups, the immunolabelling intensity of GDF9 expression in primordial follicles was decreased compared to other stages of follicular development in the granulosa cells and the oocyte cytoplasm. However, GDF9 expression was intense during the follicular development towards the primary, secondary and tertiary follicle stages (Figure 4). The increase in the GDF9 expression that was observed from primordial follicles (control, 18.86±4.03; vehicle, 19.17±3.22) towards tertiary follicles (control, 45.44±5.81; vehicle, 45.18±4.92) was statistically significant (p<0.05 and p<0.05, respectively) (Table 2, Figure 3c).

GDF9 immunoreactivity was determined in the granulosa cells and the oocyte cytoplasm of follicles in all stages of development (Figure 4). However, GDF9 immunoreactivity (PCOS, 27.73±8.43) was significantly reduced (p<0.05) compared to the other groups (control, 33.72±11.22; vehicle, 33.95±10.75; Table 1, Figure 3a). We observed that primordial follicles (PCOS, 19.83±5.51) had similar (p=0.775) immunolabelling intensity of GDF9 when compared to other groups (control, 18.86±4.03; vehicle, 19.17±3.22). Primary follicles and secondary follicles have exerted significantly increased (p<0.05) immunoreactivity compared to primordial follicles. However, the immunolabelling intensity of primary and secondary follicles decreased (p<0.05) in the PCOS group compared to the immunolabelling intensities of primary and secondary follicles of other groups. The immunolabelling intensity of tertiary follicles (PCOS, 32.63±9.17) was less when compared to other groups (control, 45.44±5.81; vehicle, 45.18±4.92) and this decrease was statistically significant (p<0.05) (Table 2, Figure 3c).

### Immunolabelling of BMP15

**Figure 5a-l:**
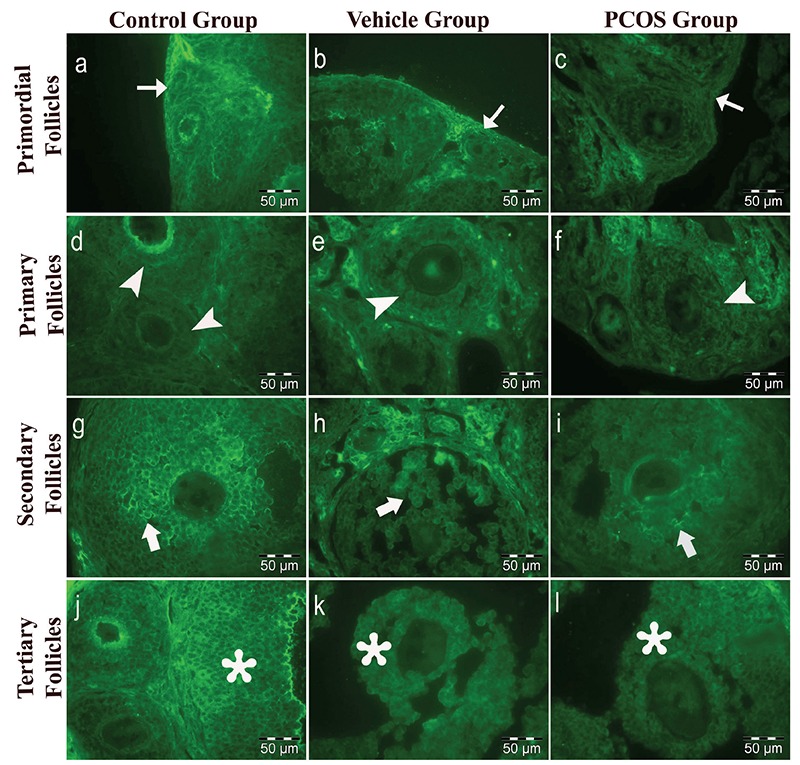
Immunolabelling intensity of BMP15. Control group: a, d, g, j; vehicle group: b, e, h, k; and PCOS group: c, f, i, l. Labelling within a-c: primordial (thin arrow); d-f: multilaminar primary (arrowheads); d-i: secondary (thick arrow) and j-l: tertiary follicles (asterisk) (x1200, FITC).

BMP15 immunoreactivity in the control group (31.12±11.05) and the vehicle group (29.99±10.72) was similar (Table 1, Figure 3b). The BMP15 expression increased during follicular development (Figure 3d, Figure 5).

In the PCOS group (24.85±7.03), BMP15 immunoreactivity in the follicular developing stages was significantly decreased (p<0.05) compared to the control group (31.12±11.05) and the vehicle group (29.99±10.72; Table 1, Figure 3b). There was prominent BMP15 immunoreactivity in the granulosa cells and in the oocyte cytoplasm of follicles. Immunolabelling intensity of BMP15 increased (p<0.05) in primary (23.69±2.89), secondary (28.94±4.47) and tertiary follicles (31.08±4.32) according to the primordial follicles (15.69±3.24). However, immunolabelling intensity of primary, secondary and tertiary follicles decreased (p<0.05) in the PCOS group compared to other groups (Figure 3d, Figure 5). This decrease in immunolabelling intensity of the BMP15 was statistically significant (Table 3).

### Ultrastructural study results

**Figure 6a-i:**
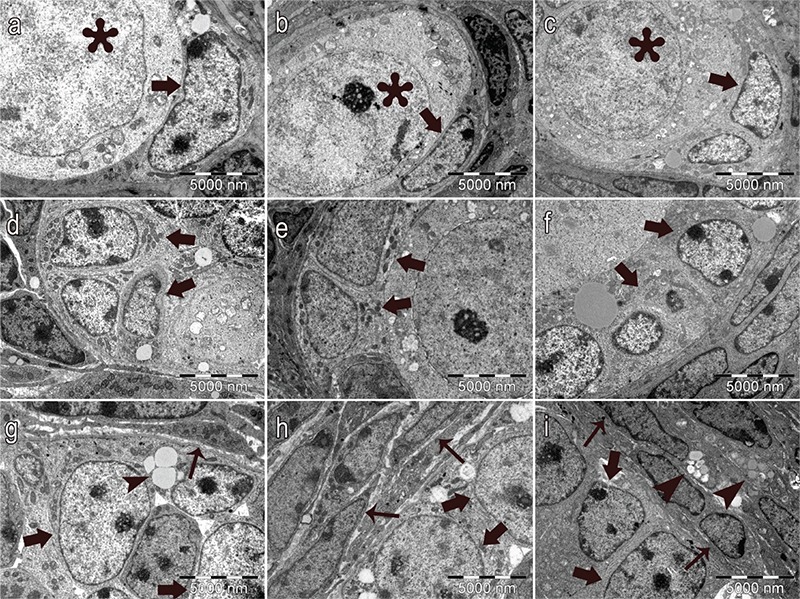
Normal structure of oocytes (asterisk) and pregranulosa cells (arrows) in primordial follicles: control group (a), vehicle group (b) and PCOS group (c). Normal structure of granulosa cells (arrows) in primary follicles: control group (d), vehicle group (e) and PCOS group (f). Normal structure of granulosa cells (thick arrows) and the surrounding Theca cells (thin arrows), lipid vacuoles (arrowheads) in multilaminar primary follicles, control group (g), vehicle group (h) and PCOS group (i) (x7500, uranyl acetate-lead citrate).

**Figure 7a-d:**
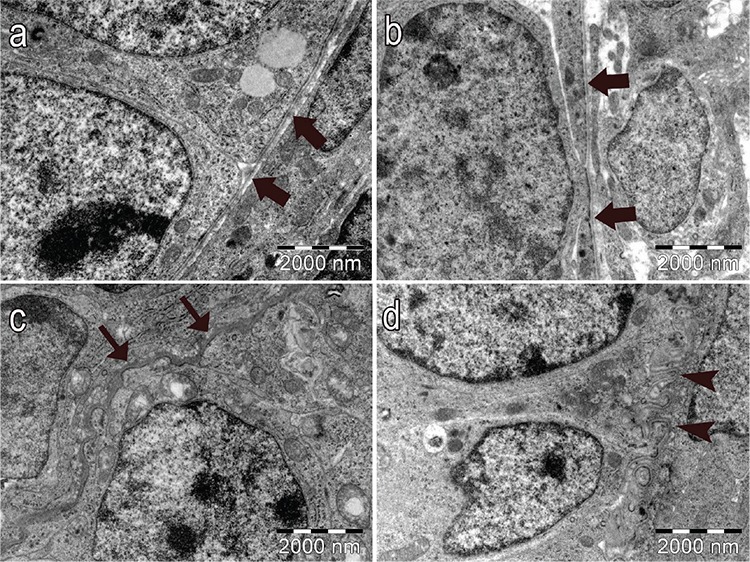
Normal structure of the basement membrane of granulosa cells (thick arrows) in the control group (a) and vehicle group (b). Thickening (thin arrows) (c) and irregularities (arrowheads) (d) in the basement membrane of granulosa cells in multilaminar primary and secondary follicles in PCOS group (x15000, uranyl acetate-lead citrate).

**Figure 8a-d:**
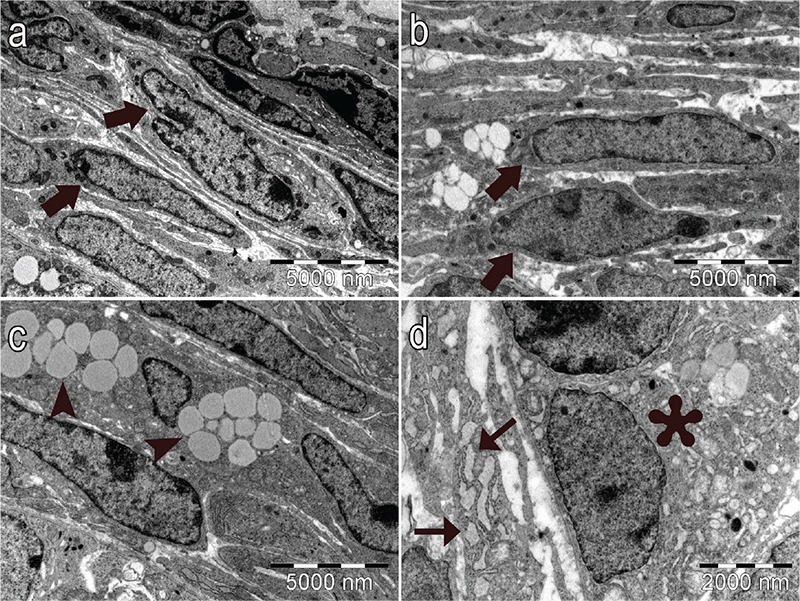
Normal structure of Theca cells (thick arrows) in secondary follicles in control group (a) and vehicle group (b) (x7500). In the PCOS group, increased lipid vacuoles in the Theca cells (arrowheads) (c) (x7500), expansion of the granular endoplasmic reticulum (thin arrows) (d) and Golgi cisternae in granulosa cells (astrerisk) (d) in the secondary follicles (x15000) (uranyl acetate-lead citrate).

**Figure 9a-f:**
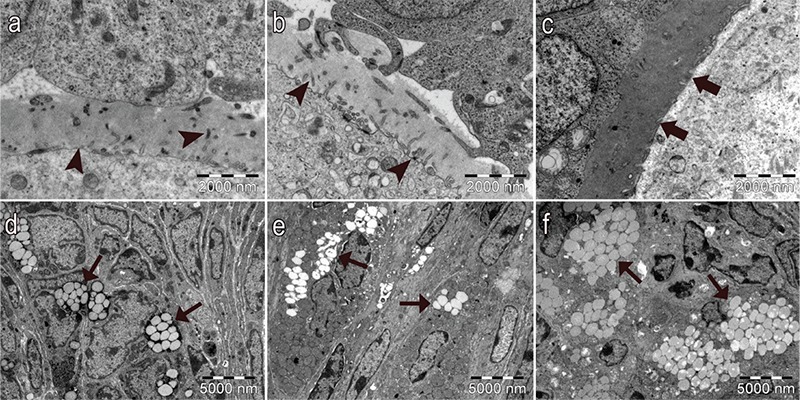
Normal structure of the ZP in control group (a) and vehicle group (b). Numerous cytoplasmic extension of the oocyte and granulosa cells (arrowheads) in ZP of control group (a) and vehicle group (b) (x15000). The electron density changes of the ZP and decreased cytoplasmic extensions of the oocyte and granulosa cells (thick arrows) in the ZP of PCOS group (c) (x15000). Lipid vacuoles in normal ovarian stromal cells (thick arrows) in the control group (d) and vehicle group (e). Increased lipid vacuoles (arrowheads) in the ovarian stromal cell cytoplasm in PCOS group (f) (x5000) (uranyl acetate-lead citrate).

In control and vehicle groups, the structure of the oocyte (Figure 6a, 6b, respectively), the granulosa cells of the follicles (Figure 6d, 6e, respectively), the basement membrane of granulosa cells (Figure 7a, 7b, respectively), the Theca cells surrounding the granulosa cells (Figure 8a, 8b, respectively) and the zona pellucida (ZP; Figure 9, respectively) had normal structure. A few lipid vacuoles were observed in some granulosa cells (Figure 6g).

**Figure 10a-d:**
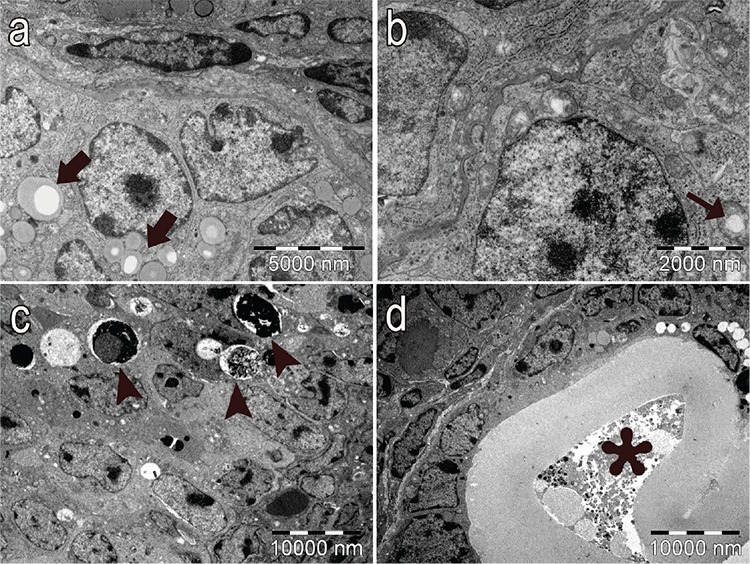
In the PCOS group, increased lipid vacuoles (thick arrows) (a) (x7500) and vacuolisation in mitochondria (thin arrows) in granulosa cells of pre-antral follicles (b) (x15000). Apoptotic granulosa cells (arrowheads) (c), follicular cystic structure surrounded by a single layer of granulosa cells and degenerated oocytes (asterisk) (x3000) (uranyl acetate-lead citrate).

In the PCOS group, the structure of the follicles was normal in the early stages of folliculogenesis (primordial and unilaminar primary follicles; Figure 6). Oocytes and pre-granulosa cells in primordial follicles were normal (Figure 6c). The basement membrane of granulosa cells had irregularities, ondulation and thickening (Figure 7c, 7d). Increased lipid vacuoles were determined in the Theca cells of the multilaminar primary follicles (Figure 8c). Granular endoplasmic reticulum cisternae in the Theca cells and the Golgi cisternae in granulosa cells of some secondary and tertiary follicles were dilated (Figure 8d). It has been shown that the ZP that surrounded oocytes was found to be more dense in the PCOS group compared to other groups. The cytoplasmic extensions of granulosa cells and oocytes in the ZP decreased prominently (Figure 9c). Additionally, lipid vacuoles in ovarian stromal cells were enhanced in the PCOS group (Figure 9f) compared with the control (Figure 9d) and vehicle groups (Figure 9e). Vacuolisation in mitochondria and increased lipid vacuoles in granulosa cells of pre-antral follicles were determined (Figure 10a, 10b, respectively). We have observed that apoptotic bodies detected in granulosa cells increased in some of the multilaminar primary and secondary follicles (Figure 10c). In addition, multiple follicular cystic structures have been detected in ovarian stroma at different stages. Degenerated oocyte particles have been observed in some cysts. (Figure 10d).

## DISCUSSION

The most common reason of anovulation is a hormonal imbalance that can cause the symptoms of PCOS (3,16). According to histological evaluation in PCOS, the number of primordial follicles in the ovary was not distinct from the ones in normal ovaries, but the number of primary and secondary follicles was shown to be significantly higher than in a normal ovary (17). Nevertheless, the autocrine-paracrine factors in the follicular microenvironment that showed variability in certain stages of folliculogenesis remains uncertain. Therefore, in our study we intended to clarify which stage of folliculogenesis in PCOS showed GDF9 and BMP15 expression changes.

Factors secreted by the oocyte are essential for follicular development and oocyte maturation (4,10). Previous studies indicate that oocyte-derived GDF9 and BMP15 support the follicle growth in the pre-antral and antral follicle stages, and they also play a role in the regulation of oocyte development and maturation (18,19). Zhao et al. (13) showed that the expression of GDF9 decreased in cumulus granulosa cells from patients with PCOS after ovulation induction, whereas the expression of BMP15 showed no significant distinction between the control and patients with PCOS. In another study, the expression levels of both GDF9 and BMP15 were decreased in metaphase II oocytes obtained from patients with PCOS after ovulation stimulation (14). A recent study demonstrated that the expression levels of GDF9 and BMP15 proteins decreased in the early stages of folliculogenesis (primordial, primary and secondary follicles) and then reached the highest levels in the late stages of folliculogenesis (graafian follicles) in unstimulated ovarian tissues from PCOS patients (15). The differences between studies can be potentially associated with the use of different methodologies, the sample size used in the experiments and the applied induction protocols. In this study, the expression levels of both GDF9 and BMP15 were decreased in the early stages of folliculogenesis, which was consistent with Wei et al. (15); however, we have found the expression of the 2 factors also decreased in the late follicular phase.

Previous studies have defined ultrastructural changes in follicular cells in PCOS (20,21,22); however, prior to the present study, no study identifying which stages of folliculogenesis exhibit these ultrastructural changes in PCOS had existed. In this study, we showed irregularities and thickening and ondulation in the basement membrane of granulosa cells in most of the multilaminar and secondary follicles in the PCOS group. Granulosa cells, which have an irregular basal lamina, showed an increase in lipid vacuoles. This finding is supported by a similar study conducted by Irving-Rodgers et al. (21). According to their results, they observed irregularities in the basal lamina of granulosa cells in some of the pre-antral follicles and antral follicles obtained from healthy cattle and reported a lack of maturation in oocytes in these follicles. In addition, they have also reported the deficiency in the expansion of the antrum and the proliferation of granulosa cells in follicles with irregular basal lamina and increase in atresia. They stated that the irregularities within the basal lamina can be associated with factors such as GDF9 and BMP15 in the follicular microenvironment. Our *in vivo* study shows that a decrease in the expression levels of GDF9 and BMP15 from the primary follicle stage towards the tertiary follicle stage. These results suggest that a decrease in GDF9 and BMP15 expression at the primary follicle stage changed the granulosa cell basal lamina structure, which then caused changes in granulosa cell function and structure at this particular stage. As in all cells, the basal lamina has an important role in the differentiation and proliferation of granulosa cells and in molecular interactions with neighbouring cells (21,23). Structural changes observed in the basal lamina of granulosa cells can disrupt the interaction between the microenvironment and the granulosa cells and can further lead to disruption in follicular development and the follicular pathological processes in PCOS.

GDF9 and BMP15 have anti-apoptotic effects. It has been reported that GDF9 suppressed apoptosis of granulosa cells and follicular atresia transition in the antral stage. It has been shown that BMP15 leads to the reduction in cumulus cell apoptosis (24). Consistent with the results of this study, we have found that apoptotic granulosa cells increased in the early follicular phase in the PCOS group. The reduction in these factors in PCOS, especially in the early follicular phase, suggests that this decrease causes follicles to enter into atretic processes instead of follicle development and can also activate the apoptotic pathway. Further studies are needed to explain the relationship between apoptosis and GDF9 and BMP15 in PCOS.

ZP which is synthesized by both granulosa cells and oocytes is essential for oogenesis, fertilization and preimplantation development. (25,26). Meczekalski et al. (27) showed that ZP4 expression among patients with PCOS with a regular cycle was highest. They claimed that the correlation between ZP4 expression and PCOS with a regular cycle was a result of the existance of mature follicles. In our study, we observed that the ZP has a dense structure in some of the antral follicles in the PCOS group. We thought that certain mechanisms in PCOS cause changes in the expression of ZP glycoproteins that may lead to the deterioration in ZP structure and function. We plan to elucidate molecular mechanisms underlying these expression changes in ZP glycoproteins in the pathogenesis of PCOS.

In conclusion, our study suggests that the reduction of GDF9 and BMP15 expression starting from the primary follicle stage can cause follicular development disorders and insufficient oocyte maturation that can further lead to subfertility or infertility. Further studies on the role of GDF9 and BMP15 in the process of follicle development and oocyte meiotic maturation will provide more information about the mechanisms that regulate these factors in the pathogenesis of PCOS.

## Figures and Tables

**Table 1:**
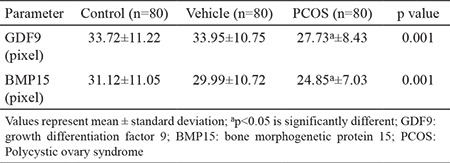
The immunolabelling intensity of GDF9 and BMP15 between groups

**Table 2:**
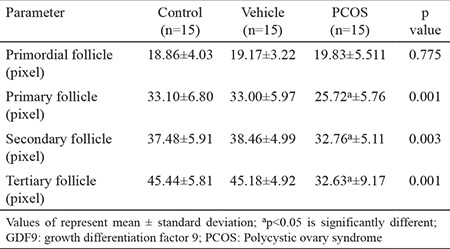
The immunolabelling intensity of GDF9 in stages of follicular development

**Table 3:**
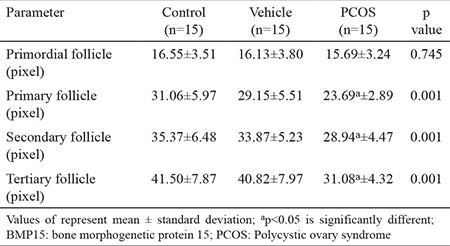
The immunolabelling intensity of BMP15 in stages of follicular development
